# Effects of *Enterobacter cloacae* extract, selenium nanoparticles and methyl jasmonate on shoot liquid cultures of *Sarcocornia fruticosa* under salinity stress

**DOI:** 10.1186/s12870-024-05988-4

**Published:** 2025-01-11

**Authors:** Fathia Salem, Raoufa Abdel Rahman, Amel Tammam

**Affiliations:** 1 Biology and Geology Department, Faculty of Education, Alexandria University, Alexandria, Egypt; 2 Department of Pharmaceutical Bioproducts, Genetic Engineering Institute, City of Scientific Research and Technology Applications, Alexandria, Egypt; 3 Department of Botany and Microbiology, Faculty of Science, Alexandria University, Alexandria, Egypt

**Keywords:** Bacterial extract (BE), MeJA, PTC, *Sarcocornia fruticosa*, SeNPs

## Abstract

**Background:**

The in vitro propagation of halophytes is innovative perspective for sustainable agriculture, conservation of natural plants and essential raw materials for industry due to increasing soil salinization and decreasing freshwater availability. *Sarcocornia fruticosa*, a halophytic plant, may hold promise for biosaline production systems and achieve bioactive products. Understanding the salt tolerance mechanisms of halophytes through elicitors can enhance the production of secondary metabolites, such as phenolics and flavonoids, under saline environment. This study aimed to evaluate the effects of NaCl salinity (700 mM and 1000 mM) on *Sarcocornia fruticosa* shoot cultures and assess the influence of different elicitors-*Enterobacter cloacae* extract (BE), selenium nanoparticles (SeNPs) and methyl jasmonate (MeJA) -on the plants growth, physiological and biochemical responses, and isorhamnetin production.

**Methodology:**

Shoot cultures were grown under controlled conditions with two concentrations of NaCl, alone and in combination with BE (0.5%), SeNPs (100 ppm), or MeJA (50 µM). Growth parameters, photosynthetic pigments, ion accumulation, osmolyte content, oxidative stress marker, enzyme activity, phenolic compound levels, and isorhamnetin production were analyzed to determine the impact of salinity and elicitor treatments on *S. fruticosa* for 14 days.

**Results:**

*Sarcorcocnia fruticosa* exhibited better tolerance up to 700 mM than 1000 mM NaCl, as evidenced by higher dry weights, chlorophyll a/b ratios, and enhanced osmolyte and antioxidant contents. Elicitation both saline cultures with BE and SeNPs improved growth mostly by increasing biomass, pigment contents, K^+^/Na^+^ ratios, and reducing lipid peroxidation, however, MeJA reduced the biomass mainly by increasing MDA and Na^+^ ion accumulation. In contrast, application of all elicitors stimulated the production of phenolic compounds and isorhamnetin, as well as BE can contribute for increasing resistance of *S. fruticosa* to stressful conditions.

**Conclusion:**

This study demonstrated that PTC techniques and appropriate elicitors can optimize halophyte propagation and secondary metabolite production under saline conditions. The findings suggest that BE and SeNPs significantly enhanced the growth and biochemical resilience of *S. fruticosa* under salinity stress, with a notable increase in isorhamnetin production. MEJA.

## Introduction

In recent decades, soil salinization has become a major issue in Egypt, affecting agricultural practices and endangering environmental sustainability and global food security [1]. Cultivating economically valuable halophyte plant species in marginal lands or croplands that are naturally salty is one efficient way to address soil salinity [2]. Halophytes are considered “smart plants” in saline environments since they can tolerate extremely saline conditions. This is due to their array of adaptive mechanisms include ions sequestration, osmotic adjustment, production of enzymatic and non-enzymatic antioxidant responses, and genetic control [3]. Halophytes play significant roles in protecting coastal regions and serve as relevant experimental models for inspection salt tolerance systems in a world where salinization [4].

*Sarcocornia fruticosa* is a euhalophytic succulent plant belonging to the family Amaranthaceae. It can grow in highly saline environments such as salt marshes, sand dunes and soil depression. *Sarcocornia* (Scott) is similar to *Salicornia* (L.); and the related genus *Arthrocnemum* Moq, both genera are conventionally named glassworts, and their fleshy shoots are commercialized under their traditional names. However, *Sarcocornia* species are perennials, Salicornia species are annuals. In more recent taxonomic revisions, all *Sarcocornia* species were merged under *Salicornia* L [5]. *Sarcocornia* species have great economic potential in various biotechnology sectors, owing to their nutritional, organoleptic, and medicinal properties [6]. They show various biological effects including antioxidant potential, anticancer, antidiabetic and immunomodulatory which derives from its secondary metabolites [7].

Recently, higher salt concentration induces accumulation highest content of total phenols and total flavonoids which correlated positively with the antioxidant activity in *Sarcocornia* species [8]. Among them, flavonol glycosides, especially quercetin-3-glucoside (Q3G) and isorhamnetin-3-glucoside (IR3G), have been regarded as the principle substances responsible for the observed biofunctional activities in *Sarcocornia* [9]. Isorhamnetin-3-glucoside (IR3G) in the form of aglycones (isorhamnetin) is known to be more efficiently act as bioactive molecules than flavonol glycosides [10]. The production of secondary metabolites is affected directly by various biochemical parameters such as the activity of precursor metabolites and key enzymes that involved in specific metabolic pathways. For instance, phenylalanine ammonia-lyase (PAL) is crucial enzyme in the phenylpropanoid pathway, leading to flavonoid production [11]. As well, salinity can trigger the production of secondary metabolites as protective mechanisms. According to recent studies, the elevation of reactive oxygen species (ROS) can activate signaling pathways that enhance the synthesis of phenolic compounds [12]. Research indicates that optimal nutrient levels can enhance the metabolic processes responsible for synthesizing these compounds [13]. However, little attention has been given to halophytic species found in Egypt, and there have been no reports on the characterization of their bioactive compounds and antioxidant activity.

Unfortunately, there is limited information available about profitable commercial farms of *Sarcocornia fruticosa* that can produce high-quality plants with consistent amounts of essential compounds. Currently, Plant biotechnology is a promising tool to enhance halophyte sustainability and an exciting area of research that may play an important role in the production of new crops, novel bioactive metabolites, conservation of natural resources, and reducing environmental and human impacts on agriculture [14].

In general, PTC may help to overcome the global crises of agricultural and horticultural production, desertification, and salinization [15]. Moreover, this technique provides a controlled, uniform environment, particularly at the cellular level under adverse environmental conditions [16]. Plant tissue cultures (PTCs) can be used to enhance the multiplication of halophytic species, propagate endangered species, and synthesis of bioactive compounds; however, only a small number of halophytes are used in tissue culture applications [17]. Various types of PTCs are employed, including callus cultures, organ cultures, suspension cultures, embryo cultures, anther cultures, protoplast cultures, and hairy root transformations [18]. Shoot culture is a particularly interesting alternative system compared to callus or cell suspension culture for secondary metabolite production and study of physiological and biochemical behaviors [19]. While molecular biology is powerful tool for breeding halophytes, they can be slow, costly, and sometimes fail to achieve desired goal. In contrast, the cultivation of halophytes using PTC techniques offers an efficient and cost-effective solution for their breeding [20].

Previous studies about halophyte species have demonstrated greater salinity tolerance as *Sesuvium portulacastrum* shoots [21]. Other studies were revealed the crucial role of NaCl in efficient in vitro propagation of *Salicornia brachiata* [22] and *Limoniastrum monopetalum* [23]. However, in vitro studies of salinity tolerance in halophytic plant species remain relatively rare [24], and comparative studies using both tissue cultures and whole plants grown in substrate are even scarcer [25]. Elicitors are among the various biotechnological approaches for the enhanced production of novel bioactive compounds [26].The application of elicitors to culture media as potential for mitigating the effects of stress on plant growth and development as well as challenges and opportunities as tools to stimulate secondary metabolite production, such as methyl jasmonate (MeJA), chitosan, metal ions, nanoparticles, bacteria, and fungi is considered an enhancement strategy [27]. The main objective of this study was to employ in vitro shoot liquid culture as a model system to halophytic plant *Sarcocornia fruticosa* and examine the effect of biotic and abiotic elicitors (rhizospheric bacterial extract (BE), selenium nanoparticles (SeNPs) and methyl jasmonate (MeJA) on biomass and isorhamnetin accumulation under NaCl treatments. This paper also focuses on effect of these elicitors on specific biochemical parameters such as the concentration of photosynthetic pigments, different monovalent and divalent ions, osmolytes contents, oxidative stress marker, phenolic compounds, and the production of desired bioactive isorhamnetin.

## Materials and methods

### Experimental material

Healthy branches of *Sarcocornia fruticosa* were collected from El-Hammam salt marsh (29°34`27.9” N, 30°55`15.7” E) in the Western Mediterranean Coastal Zone, Alexandria governorate, Egypt. Collected branches were verified by Dr. Sania kamal from Faculty of Science, Alexandria University, and a voucher specimen was deposited in the herbarium of department of botany and microbiology, Faculty of Science, Alexandria University, deposition number 4114. Shoots were sterilized by immersion in 70% ethanol for 1 min, followed by 30% commercial bleach Clorox (5.25% sodium hypochlorite) containing few drops of Tween 20 for 30 min and finally rinsed with sterile distilled [28]. For propagation, shoots were cultured in double-strength Murashige and Skoog (DMSS) medium [29] containing 2% NaCl, 3% sucrose, and 0.7% agar, without plant growth regulators according to [30] with pH adjusted to 5.7 before autoclaving. Sodium chloride (NaCl) was prepared at concentrations of 700mM/L and 1000mM/L. *Enterobacter cloacae* 13159_1 CHB was use as bacterial extract (BE) according to [31] at concentration 0.5%. Selenium nanoparticles (SeNPs) were characterized by Scanning Electron Microscopic (SEM) and X-ray diffraction (XRD) analysis. A sterilized 100 ppm SeNPs suspension was prepared using MilliQ water and ultrasonicated for 15 min to ensure homogeneity. *Enterobacter cloacae* and SeNPs were provided from the City of Scientific Research and Technology Applications. Methyl jasmonate (Sigma Aldrich, USA) was prepared as 0.1 M stock solution and diluted to 50 µM, and then sterilized using 0.22- µm nylon syringe filters. The selected concentrations of NaCl, *Enterobacter cloacae* (BE), SeNPs and MeJA were determined after preliminary screening for various concentrations which based on previous literature. The selected elicitors concentrations were the most effective for cultures growth. NaCl ( 320mM, 700mM, 1000mM); BE (0.5%, 1% and 2%); SeNPs (50 ppm and 100 ppm); and MeJA (50 µM, 100 µM to 200 µM).

### Plant culture and treatments

**Fig. 1:**
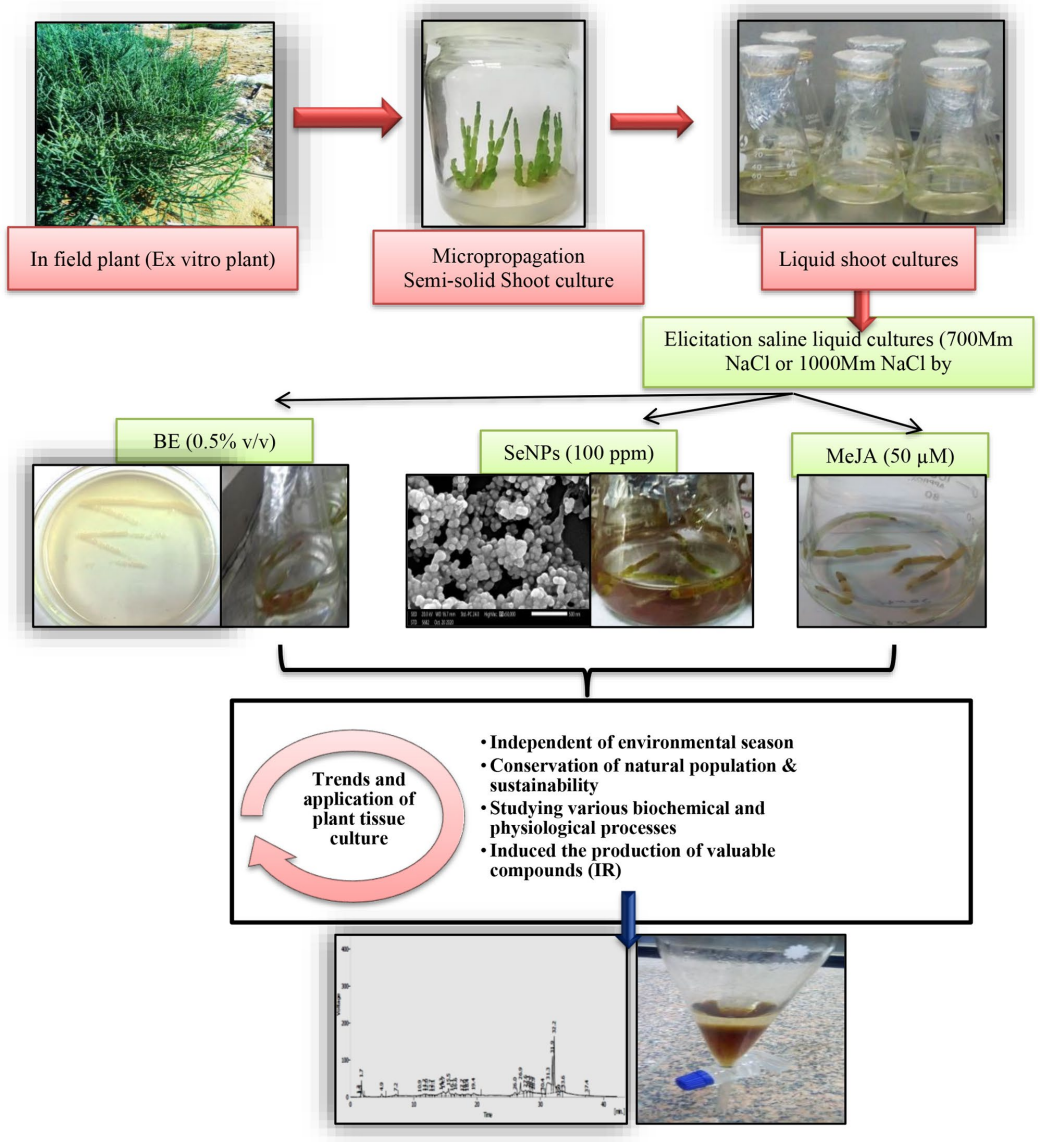
The experimental process of *Sarcocornia fruticosa* shoots liquid culture

The outline of this investigation was presented in Fig. 1. Sterilized branches were dissected into nodal segments (3–4 nodes) as explants and cultured on semi-solid double strength MS medium-free hormone containing 2% NaCl (DMSS) for 30 days. The growing lateral branches were dissected into nodal segments and four segments were transferred to Erlenmeyer flasks (100 ml) containing 40 ml of liquid DMSS-free agar as a subculture. After 7 days, shoot segments were performed as replicate cultures that included: (1) Control (DMSS; double strength MS containing 2% NaCl without addition of any elicitors), (2) 700mM NaCl (double strength MS (DMS) containing 700mM NaCl, (3) 1000mM NaCl (double strength MS (DMS) containing 1000mM NaCl), (4) 700mM NaCl /0.5% BE (double strength MS containing 700mM NaCl and elicited with 0.5% BE), (5) 700mM NaCl/SeNPs (double strength MS containing 700mM NaCl and elicited with 100 ppm of SeNPs, (6) 700mM NaCl/50µM MeJA (double strength MS containing 700mM NaCl and elicited with 50µM of MeJA, (7) 1000mM NaCl /0.5% BE (double strength MS containing 1000mM NaCl and elicited with 0.5% BE), (8) 1000mM NaCl / SeNPs (double strength MS containing 1000mM NaCl and elicited with 100 ppm of SeNPs) and (9) 1000mM NaCl /50 µM MeJA (double strength MS containing 1000mM NaCl and elicited with 50 µM of MeJA). All treated flasks were randomly arranged, with ten replicates for a total of 90 flasks, which incubated in a rotary shaker at 120 rpm and 24 ± 2 ˚C under 16/8 h light/dark cycle. All cultures were harvested after 14 days of the treatments, since the growth and physiological performance in cultures were determined.

### Selenium nanoparticles characterization

Selenium nanoparticles were characterized by Scanning Electron Microscopic (SEM) and X-ray diffraction (XRD) analysis.

### Detection of selenium nanoparticles in shoot tissue

Accumulated SeNPs in segments were analyzed by EDX spectroscopy. Nodal explants exposed to 100 ppm of SeNPs were rinsed with sterile DDW and immersed immediately in 4F1G in phosphate buffer solution (PH 7.2) at 4 °C for 3 h. Specimens were then post fixed in 2% OSO_4_ in the same buffer at 4 °C for 2 h. Samples were washed in the buffer and dehydrated at 4 °C through a graded series of ethanol. The dehydrated tissues were then processed for EDX analysis.

### Culture growth parameters

To measure biomass production, shoots of *S. fruticosa* were harvested and the fresh weight (FW) was immediately determined. Part of the shoot segments were placed into an oven at 50 °C until constant weighed to record dry weights. The water content percentage of each sample was determined according to the following formula: WC (%) = [(FW − DW)/FW] × 100 [32].

### Biochemical analysis

#### Photosynthetic pigments assay

Photosynthetic pigments chlorophyll a, b, total and carotenoids were determined in treated fresh shoot cultures as described by [33, 34].

#### Mineral element analysis

Ions were extracted from dry shoot cultures according to [35]. The content of sodium (Na^+^), potassium, (K^+^) calcium (Ca^2+^) and magnesium (Mg^+ 2^) in shoot samples were quantified with Inductively Coupled Plasma-Optical Emission Spectroscopy (ICP-OES; Agilent 5100 VDV, USA) as mg/g. DW.

#### Determination of soluble sugars (SS)

Soluble sugar content was extracted from dry tissues according to the method by [36] and determined by the method described by [37]. Absorbance was measured at 490 nm. The amount of sugar was calculated as mg/g DW.

#### Determination of soluble protein (SP)

The concentration of soluble proteins was extracted as described by [38]. Soluble protein was determined according to the method described by [39, 40]. Concentrations of soluble protein plant tissue are expressed as mg/g DW.

#### Determination of free proline

Proline was estimated according to the method described by [41]; Proline was employed in different concentrations (10–100 µg) to draw a standard curve from which the amount of proline was calculated as µmoles proline/g. FW.

### Estimation of malondialdehyde (MDA) content

Lipid peroxidation in shoot segments was determined by estimating the malondialdehyde (MDA) according to the method [42]. The non-specific absorbance at 600 nm was subtracted from the maximum absorbance at 532 nm for MDA measurement. The concentration of MDA was calculated using an extinction coefficient of 155 mM/L^–1^ cm^–1^.

### Estimation of superoxide dismutase (SOD, EC 1.15.1.1) activity

The extraction of enzymes was carried out following the method [43]. The activity of SOD was assayed based on the inhibition of the photochemical reduction of nitro blue tetrazolium (NBT) by superoxide radicals to formazan according to method [44]. One SOD unit was defined as the amount of enzyme required for 50% inhibition of NBT reduction at 560 nm.

### Estimation of ascorbate peroxidase (APX, EC 1.11.1.11) activity

Ascorbate peroxidase (APX) was assayed as described by [45]. The decrease in absorbance at 290 nm for 1 min was recorded and the amount of ascorbate oxidized was calculated from the extinction coefficient (Δε) 2.8 mM^–1^cm^–1^. One unit of APX activity was defined as the amount of enzyme oxidizing one mM ascorbate per minute.

### Estimation of guaiacol peroxidase (GPX, EC 1.11.1.7) activity

Guaiacol peroxidase (GPX) activity was determined according to [46]. The enzymatic reaction rate was calculated by monitoring guaiacol oxidation to tetraguaiacol at 470 nm for 5 min (Δε = 26.6 mM L^–1^cm^–1^). The specific activity of GPX was expressed as the amount of enzyme that oxidizes 1 mM of guaiacol per minute per mg protein.

### Estimation of Polyphenol oxidase (PPO, EC 1.10.3.1) activity

Polyphenol oxidase activity was determined according to the method [47] based on increasing in absorbance at 410 nm. The enzymatic reaction rate was recorded continuously at 25^°^C for 5 min. The polyphenol oxidase specific activity (PPO) expressed as mM min^− 1^ mg protein^− 1^.

### Extraction and determination of phenylalanine-ammonia lyase (PAL, EC 4.3.1.5) activity

Phenylalanine ammonia-lyase (PAL) activity was extracted following the method [48], and enzyme was assayed following the method [49]. The absorbance of trans-cinnamic acid was measured at 290 nm and the specific enzyme activity was expressed as l µM of cinnamic acid/ mg protein.

Protein in all enzymes extracts was estimated according to [50] using bovine serum albumin (BSA).

### Determination of total phenolic content

The powdered tissues of each sample were individually extracted as described by [51]. Total phenolic contents were determined according to method [52] with some modification. Aliquot of 200 µl of tissues extract was mixed to 3 ml of water and 200 µl of Folin- Ciocalteau reagent (Sigma). The test tubes were allowed to stand for 10 min, and then 1 ml of 20% Na_2_CO_3_ was added. After 20 min at 40^°^C the absorbance was measured at 750 nm. Phenolic concentration was expressed as mg gallic acid /g DW.

### Determination of total flavonoid content

The aluminum chloride colorimetric method was used for the determination of the total flavonoid content of samples according to [53]. The absorbance of the reaction mixtures was measured against blank at 415 nm. The concentration of total flavonoid content expressed as mg rutin /g of DW of segments tissue.

### Determination of flavonol content

Flavonol contents were determined through the test described by [54] with modification. Aliquot of methanolic extract 0.3 ml was added to 0.3 ml of AlCl_3_ (2 mg/ml) and 1.5 ml sodium acetate (50 mg/ml). The absorbance at 440 nm was recorded after 2.5 h of incubation. The content of flavonols was expressed as mg of quercetin /g dry weight of plant tissue (mg Q /g DW).

#### Determination of DPPH free radical-scavenging activities

The antioxidant activity of plant methanol extracts was determined by using the stable 2, 2-diphenyl-1-picryl-hydrazyl radical (DPPH) according to method [55]. The radical scavenging activity was expressed as percentage inhibition of DPPH, which calculated according to the following formula proposed by [56].

DPPH scavenging activity (%) = (A blank – A sample/ A blank) x 100, where A blank is the absorbance of the control reaction; A sample is the absorbance of the test compound.

### Isorhamnetin extraction and acid hydrolysis

Isorhamnetin (IR) was extracted with some modifications based on the method described in [57]. A total of 0.25 g of dry tissue of *S. fruticosa* shoot cultures were homogenized in 70% (v/v) methanol and stirred overnight on rotary shaker at 4 °C. After filtration, the residues were extracted for three additional times. Acid hydrolysis was done by adding 2 ml of concentrated H_2_SO_4_ solution to the extract, and the mixture was heated on a heating block at 90˚C for 60 min before cooling to room temperature. The hydrolyzed samples were filtered through No. 2 filter paper (Whatman), and combined filtrates were concentrated under vacuum at 38˚ C. The concentrate was suspended in 50 ml distilled water and partitioned with ethyl acetate three times. The supernatant (EtOAc layer) was concentrated, and the residue was dissolved in 2 ml analytical grade MeOH for HPLC analysis. The solution was filtered through a 0.45 μm Millipore membrane.

#### High-performance liquid chromatography (HPLC) of isorhamnetin

Flavonol isorhamnetin (IR) was purchased from Sigma-Aldrich and quantified according to method describe by [58]. Filtrated samples were analyzed using a high-performance liquid chromatography (HPLC) system consisted of Agilent 1200 series HPLC apparatus (Agilent Technologies, Santa Clara, CA, USA), including high-pressure binary-gradient solvent-delivery pump, DAD (diode-array detector). Aligent C18 column (4.6 × 250 mm, 5 μm) was used for separate analysis using a linear gradient of mobile phase with O-phosphoric acid 0.25% (A)- acetonitrile (B) for 42 min starting with A: B (95:5) for 2 min, changing to A: B (90:10) for 5 min, A:B (85:15) for 3 min, A:B (80:20) for 13 min, A:B (70:30) for 5 min, A:B (50:50) for 4 min with equilibrating for 10 min. The flow rate was 1 ml/min. The injection volume for all triplicate samples and standard solutions was 10 µl and detection was conducted at wavelengths of 360 nm. Quantification relied on an external standard method; calibration curves were established for concentrations of 5–25 µg of isorhamnetin, with the equation y = 58.95x − 0.6295 (R² = 0.9974) correlating peak areas to concentration.

### Statistical analysis

The experiment was designed in a completely randomized design with at least three replicates (*n* = 3) and all data were represented as mean ± SD. The results were analyzed by comparing (F) values obtained from a one-way ANOVA by using the statistical software SPSS software (Version 16) [59] to test the effects of NaCl and its elicitation with BE, SeNPs, and MeJA on the measured variables. Levene’s test was used to investigate the homogeneity of variances of all data. Duncan’s multiple comparisons test was used to determine statistically significant differences among the treatments at 95% confidence limit (*p* < 0.05) and values denoted by the same letter are not significantly different. Microsoft Excel (2016) was used to prepare all graphs. A heat map with two cluster analyses and principle component analysis (PCA) (linear ordination technique) were conducted using R-program (R i386 3.4.3). Pearson’s correlation coefficient was analyzed by the software OriginPro, version 2022 (OriginLab Corporation, Northampton, MA, USA) to explore the correlation between all parameters under different treatments. Parameter’s rank is represented in color, where the intense red color represents the high value, while the light red to green color represents the low value.

## Results

### Characterization of selenium nanoparticles (SeNPs)

**Fig. 2:**
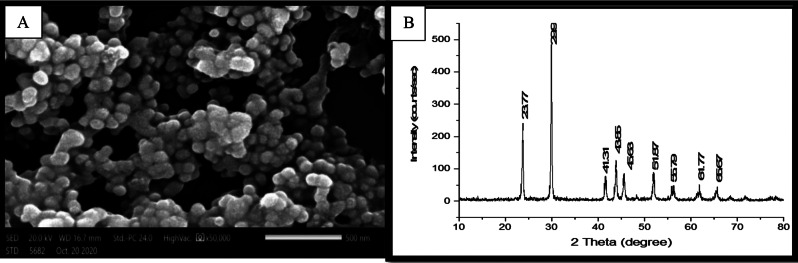
Scanning electron microscopy micrograph (magnification 50.000x) (**A**) and X-ray diffraction patterns (**B**) of selenium nanoparticles (SeNPs)

Scanning electron microscopy (SEM) revealed that the synthesized selenium nanoparticles (SeNPs) have spherically shaped and smooth surface, and with average particle size ranging from 50 to 100 nm (Fig. 2). X-ray diffraction (XRD) measurements identified several distinct peaks at 2 theta (2θ) values: 23.77, 29.9, 41.31, 43.85, 45.63, 51.87, 55.79, 61.77, and 65.67. these peaks correspond to the standard diffraction patterns of selenium, as indicated by the Joint Committee on Powder Diffraction Standards (JCPDS) no. 04-007-0333. The identified diffraction peaks suggest that the SeNPs crystallize in a hexagonal phase, with lattice constants determined to be a = 4.36 Å and c = 4.95 Å. The sharpness of these peaks indicates that the selenium nanoparticles exhibit a high degree of crystallinity, signifying that the synthesis process effectively yielded well-ordered, stable structures.

### Uptake of selenium nanoparticles by shoot tissues

**Fig. 3:**
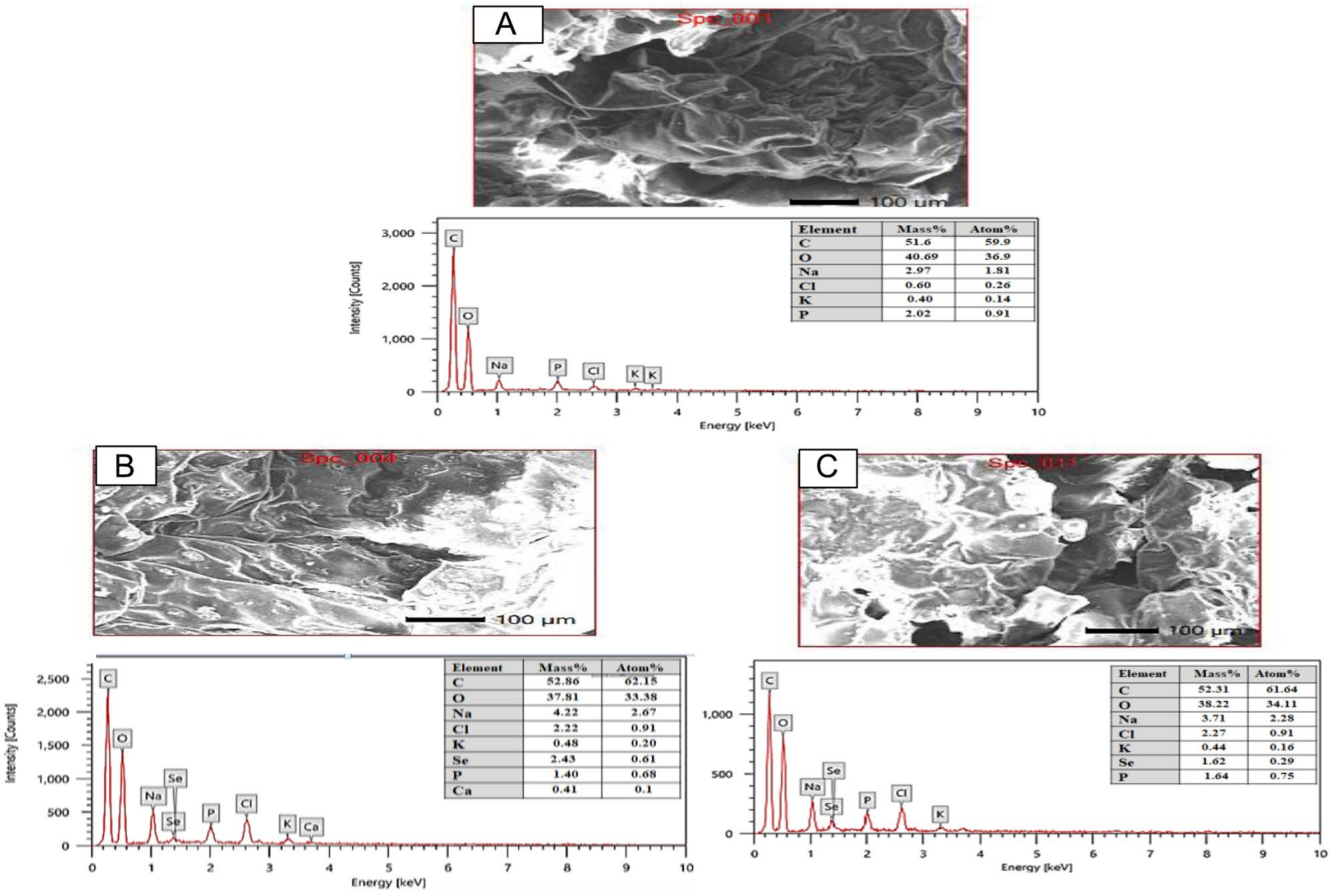
Energy dispersive X-ray (EDX) based analysis of SeNPs and their detection on the surface of *S. fruticosa* shoot after exposure to SeNPs for 14 days. (**A**) Control, (**B**) 700 mM NaCl/100 ppm SeNPs, (**C**) 1000 mM NaCl/100 ppm SeNPs

After 14 days of exposure, energy-dispersive x-ray (EDX) analyses confirmed the presence of SeNPs in the shoot tissues after the exposure times shown in Fig. 3B and C; they were not detected in the control (Fig. 3A). Other elements, such as oxygen, carbon, sodium, chlorine, phosphorus, and potassium, could have originated from the shoot tissues.

### Changes in growth parameters of *S. fruticosa* shoot cultures in response to elicitors and/or NaCl

Shoot cultures treated with 700 mM NaCl exhibited a significant increase in dry weight (DW) but WC% decreased slightly. Using double-strength MS medium (DMS) with 700mM NaCl and 0.5% BE or 100 ppm SeNPs produced a significant increase in DW. However, at 1000mM NaCl, DW increased significantly compared to the control, fresh weight (FW) and WC% were significantly inhibited (F = 4.83, Table 1). Moreover, both FW and DW improved at DMS medium contains 1000 mM NaCl, and either 0.5% BE or 100 ppm SeNPs. In contrast, significant reductions in FW, DW, and WC% were observed in saline DMS medium (700 mM or 1000 mM NaCl) treated with 50 µM MeJA (F = 12, Table 1).

**Table 1:** Effect of NaCl treatments (700mM and 1000mM) and their elicitation with bacterial extract (BE), selenium nanoparticles (SeNPs), or methyl jasmonate (MeJA) on fresh, dry weight and water content % (WC%) of *Sarcocornia fruticosa* shoot cultures after 14 days of treatments

Treatments	Fresh Weight (gm/ flask)	Dry Weight (gm/flask)	WC %
Control	1.36 ^a^ ± 0.02	0.095^d^ ± 0.03	93.0^a^ ± 0.5
700 mM NaCl	1.37 ^a^ ± 0.1	0.118 ^c^ ±0.02	91.5^b^ ±0.9
1000 mM NaCl	1.02 ^c^ ± 0.09	0.116^c^ ± 0.04	87.7^e^ ± 0.21
700 mM/0.5% BE	1.34 ^a^ ± 0.12	0.137^a^ ± 0.08	91.0^b^ ±0.5
700 mM/SeNPs	1.35 ^a^ ± 0.10	0.122^c^ ± 0.01	91.4^b^ ±0.1
700 mM/50µM MeJA	0.88 ^d^ ± 0.02	0.091^d^ ± 0.08	89.6^cd^ ± 0.9
1000 mM/0.5% BE	1.24 ^ab^ ± 0.10	0.134 ^ab^ ±0.02	89.1^d^ ± 0. 1
1000 mM/SeNPs	1.13 ^b^ ± 0.12	0.124 ^cb^ ±0.01	89.6 ^cd^ ±0.7
1000 mM/50µM MeJA	0.83 ^d^ ± 0.02	0.085^d^ ± 0.0 8	86.8^e^ ± 0.8
F	**25.230**	**4.824**	**12.000**
*P* value	**0.000**	**0.003**	**0.000**

Mean ± SD (*n* = 3). Different letters indicate statistically significant differences as evaluated by ANOVA and Duncan’s multiple comparison test for *P* < 0.05 level

### Changes in photosynthetic pigments of *S. fruticosa* shoot cultures in response to elicitors and/or NaCl

In *S. fruticosa* shoot cultures, treatment with 700 mM or 1000 mM NaCl significantly reduced (*P* > 0.05) photosynthetic pigments (Chl a, Chl b, carotenoids, and total chlorophyll), while Chl a/b ratio increased (F = 67.402, 14.271, 20.085, 19.625 respectively, Table 2). Furthermore, supplementing saline media with 0.5% BE resulted in reduction of Chl a, and increasing Chl b. While 100 ppm SeNPs boosted maximum amounts of all pigment levels. Elicitation with 50 µM MeJA decreased significantly all pigment contents in both saline cultures except Chl b at 700 mM NaCl. The Chl a/ Chl b ratio was significantly decreased in saline DMS medium (700 mM or 1000 mM NaCl) elicited with 0.5% BE, 100 ppm SeNP, or 50 µM MeJA respective to the relevant stress treatments (F = 70.517, Table 2).

**Table 2:** Effect of NaCl treatments (700mM and 1000mM) and their elicitation with bacterial extract (BE), selenium nanoparticles (SeNPs), or methyl jasmonate (MeJA) on photosynthetic pigments of *Sarcocornia fruticosa* shoot cultures after 14 days of treatments

Treatments	Ch l a (mg/g.FW)	Chl b (mg/g.FW)	Car (mg/g.FW)	Total Chlorophyll (mg/g.FW)	Chl a/ b
Control	45.95^a^ ± 0.7	34.07^a^ ± 1.3	8.05 ^a^ ± 0.4	80.02 ^a^ ±1.7	1.35^b^ ± 0.05
700 mM NaCl	42.69^bc^ ± 0.5	29.40^d^ ± 0.6	5.90 ^b^ ± 0.2	72.09 ^b^ ± 0.2	1.45^a^ ± 0.03
1000 mM NaCl	41.14 ^c^ ±1.3	29.30^d^ ± 1.7	5.32^b^ ± 1.0	71.44 ^b^ ± 2.1	1.44^a^ ± 0.04
700 mM/0.5% BE	41.53 ^c^ ± 1.0	31.98^c^ ± 0.9	5.89^b^ ± 0.9	73.51 ^b^ ± 1.8	1.29^c^ ± 0.06
700 mM/SeNPs	45.0 ^ab^ ± 0.9	33.30 ^b^ ± 0.3	7.32 ^a^ ± 0.2	78.32 ^ab^ ±1.1	1.35^b^ ± 0.02
700 mM/50µM MeJA	33.54^d^ ± 0.9	32.18^bc^ ± 0.1	2.48^d^ ± 1.0	66.03^c^ ± 1.0	1.03^d^ ± 0.03
1000 mM/0.5% BE	34.52^d^ ± 1.8	31.62^c^ ± 0.7	4.43^c^ ± 0.7	66.15^c^ ± 2.0	1.09^d^ ± 0.04
1000 mM/SeNPs	43.53^b^ ± 1.4	32.33^b^ ± 1.2	5.70^b^ ± 0.7	75.86 ^ab^ ± 1.6	1.34^b^ ± 0.04
1000 mM/50µM MeJA	29.92^e^ ± 1.0	27.48^e^ ± 1.1	2.43^d^ ± 0.1	57.41 ^d^ ± 1.6	1.09^d^ ± 0.04
F	**67.402**	**14.271**	**20.085**	**19.625**	**70.517**
*P* value	**0.000**	**0.000**	**0.000**	**0.000**	**0.000**

Mean ± SD (*n* = 3). Different letters indicate statistically significant differences as evaluated by ANOVA and Duncan’s multiple comparison test for *P* < 0.05 level

### Changes in elements contents of *S. fruticosa* shoot cultures in response to elicitors and/or NaCl

**Fig. 4:**
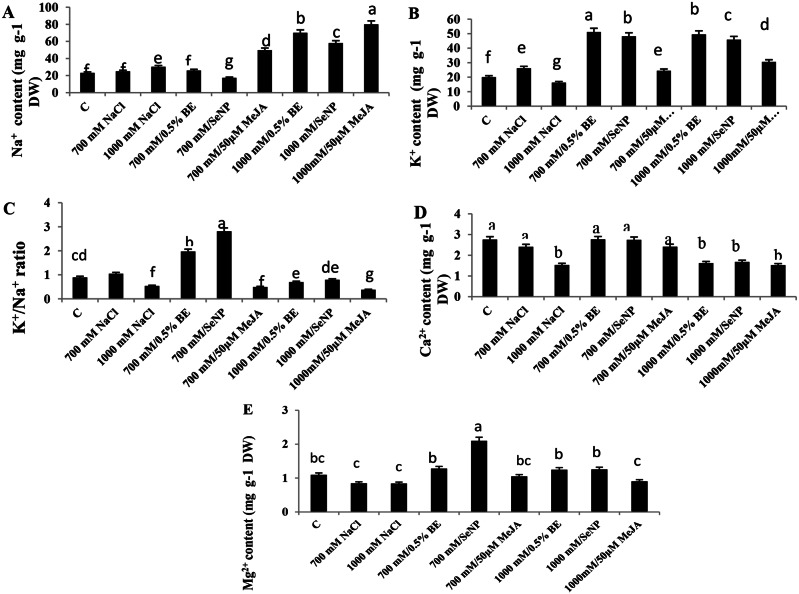
Influence of NaCl treatments (700mM and 1000mM) and their elicitation with 0.5% bacterial extract (BE), 100 ppm selenium nanoparticles (SeNPs), or 50 µM methyl jasmonate (MeJA) on elements accumulation after 14 days of treatments. (**A**) Na^+^, (**B**) K^+^, (**C**) K^+^/ Na^+^ ratio, (**D**) Ca^2+^, and (**E**) Mg^2+^ content in shoot cultures of *Sarcocornia fruticosa*. The values represented on bars are means ± standard deviation of three replicates (*n* = 3). Different letters indicate statistically significant differences between the treatments as evaluated by ANOVA and Duncan’s multiple comparison test (*P* < 0.05)

Salinity induced a higher Na^+^ uptake and diminished K^+^, Ca^2+^, and Mg^2+^ contents in shoot cultures of *S. fruticosa*, where Na^+^ accumulation increased significantly (*P* < 0.05) up to 40-fold at 1000 mM NaCl culturea medium. No substantial change in Na^+^ content was observed at 700 mM NaCl with 0.5% BE, however, a significant reduction was detected by applying 100 ppm SeNPs. Conversely, Na^+^ concentration at 1000 mM NaCl medium increased with 0.5% BE or 100 ppm SeNPs respective to stress culture. Elicitation cultures with 50 µM MeJA at either 700 mM or 1000 mM NaCl caused a significant increases in Na^+^ concentrations (F = 515.1, Fig. 4A). Potassium showed an initial increase at 700 mM NaCl but decreased significantly at 1000 mM NaCl (F = 518.494, Fig. 2B). Elicitors increased K^+^ content in the saline cultures compared to untreated stress conditions, except 50 µM MeJA did not triggered any change in K^+^ content at 700 mM NaCl medium ( Fig. 4B).

Salt treatment disrupted the Na^+^/K^+^ balance in *S. fruticosa* shoot cultures. Raising the Na^+^/K^+^ ratio was observed especially with 0.5% BE or 100 ppm SeNPs (F = 75.72, Fig. 4C). In contrast, 50 µM MeJA reduced K^+^/Na^+^ ratios by 52% and 37% at 700 mM and 1000 mM NaCl, respectively. Calcium levels were stable at 700 mM NaCl but dropped at 1000 mM NaCl (47%) related to the control (F = 8.56, Fig. 4D), while elicitation showing no significant effect respective to the relevant stress cultures. The content of Mg^2+^ significantly fell at both saline concentrations, although elicitation with 0.5% BE or 100 ppm SeNPs increased Mg^2+^ compared to 50 µM MeJA (F = 16.35, Fig. 4E).

### Change in osmolytes and oxidative stress marker of *S. fruticosa* shoot cultures in response to elicitors and/or NaCl treatments

**Fig. 5:**
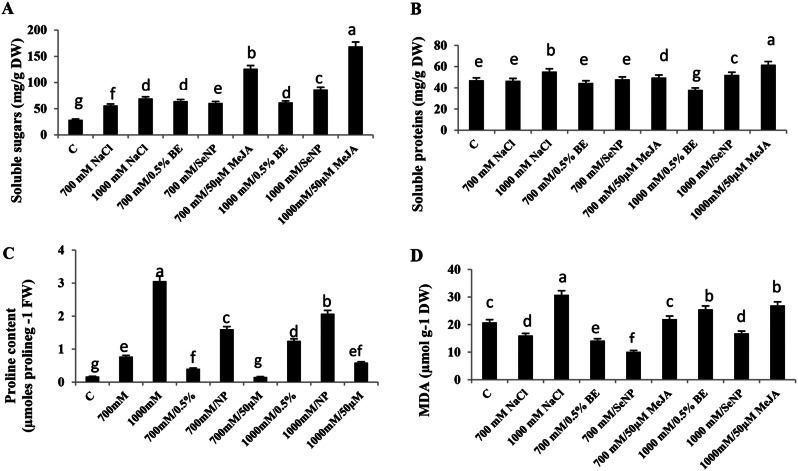
Influence of NaCl treatments (700mM and 1000mM) and their elicitation with 0.5% bacterial extract (BE), 100 ppm selenium nanoparticles (SeNPs) or 50 µM methyl jasmonate (MeJA) on compatible solutes and oxidative marker after 14 days of treatments. (**A**) Soluble sugar content, (**B**) Soluble proteins, (**C**) Proline content, (**D**) MDA content in shoot cultures of *Sarcocornia fruticosa*. The values represented on bars are means ± standard deviation of three replicates (*n* = 3). Different letters indicate statistically significant differences between the treatments as evaluated by ANOVA and Duncan’s multiple comparison test (*P* < 0.05)

Soluble sugars, proteins, and proline are vital compatible solutes for osmotic adjustment in *S. fruticosa* shoot cultures under salinity and elicitor treatments. In comparing to control, cultures exposed to 700 mM or 1000 mM NaCl showed significant (*P* < 0.05) increase in soluble sugars. Further enhancement of soluble sugar levels were observed after elicitation both saline media with 0.5% BE, 100 ppm SeNPs, or 50 µM MeJA, respective to the relevant stress culture (F = 1134.1, Fig. 5A).

Soluble proteins also increased significantly (*P* < 0.05) in 1000 mM NaCl- treated culture. Nevertheless, after elicitation, no significant differences were recorded at 700 mM, while the concentration decreased at 1000 mM. Notably, cultures treated with 50 µM MeJA achieved the highest protein levels (F = 203.099, Fig. 5B).

Proline accumulated significantly with increasing NaCl concentrations (700 mM and 1000 mM NaCl) (Fig. 5C). Shoot cultures at 700 mM supplemented with 0.5% BE or 50 µM MeJA exhibited a significant reduction in the proline content, while 100 ppm SeNPs increased proline content. In 1000 mM NaCl- treated cultures, proline decreased significantly with all elicitors (F = 548.72).

Statistical analysis of malondialdehyde (MDA) content revealed significant differences in *S. fruticosa* cultures grown in 700 mM and 1000 mM NaCl cultures compared to the control. MDA contents significantly decreased in cultures treated with 0.5% BE or 100 ppm SeNPs under both salt concentrations. However, MDA content increased significantly (*P* < 0.05) in cultures at 700 mM supplemented with 50 µM MeJA and decreased at 1000 mM with the same supplement (F = 314.91, Fig. 5D).

### Changes in enzymes activities of *S. fruticosa* shoot cultures in response to elicitors and/or NaCl

**Fig. 6:**
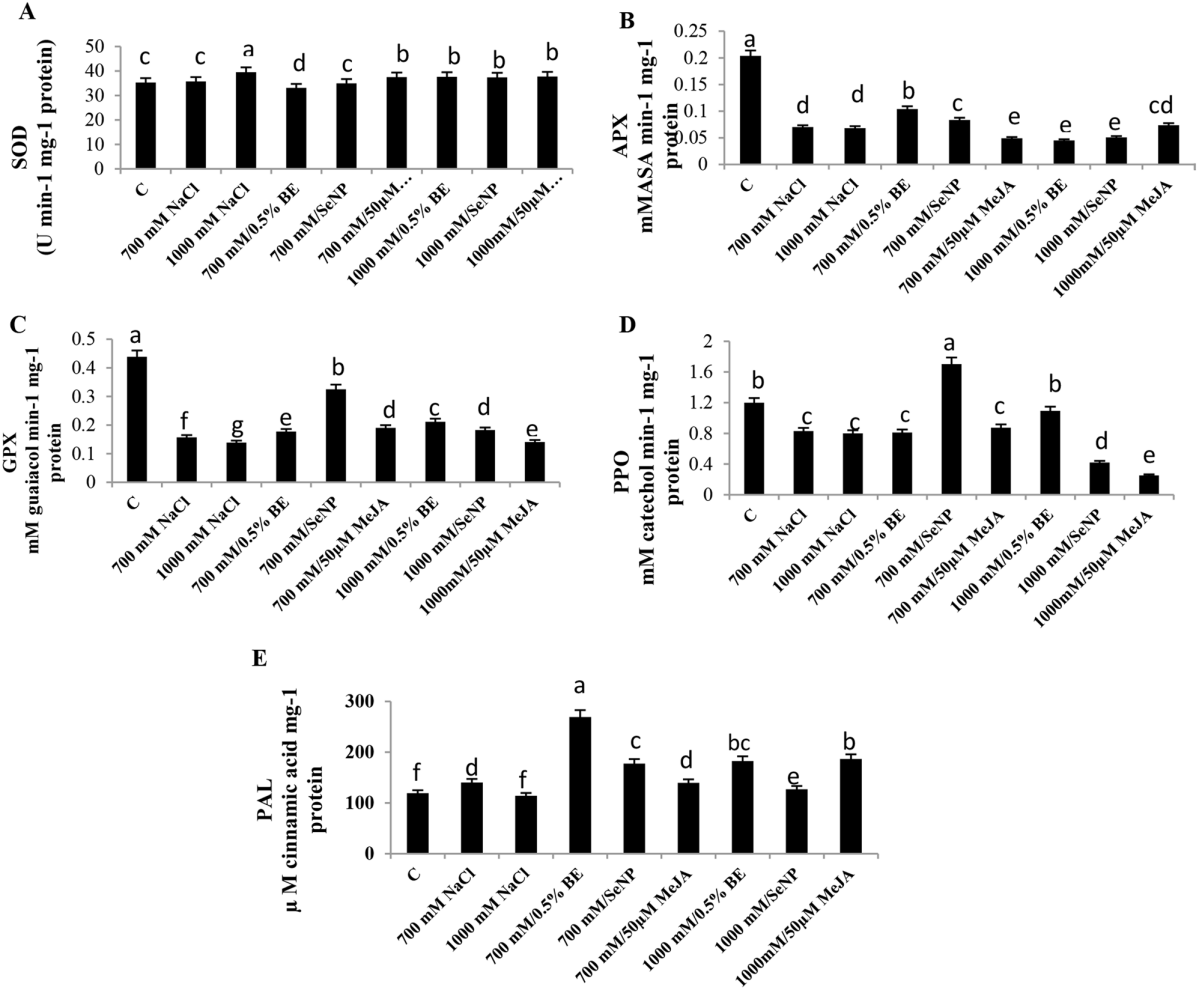
Modulation of enzyme activities in *Sarcocornia fruticosa* cultures treated with NaCl treatments (700mM and 1000mM) and their elicitation with 0.5% bacterial extract (BE), 100 ppm selenium nanoparticles (SeNPs), or 50 µM methyl jasmonate (MeJA) after 14 days of treatments. (**A**) Superoxide dismutase (SOD), (**B**) Ascorbate peroxidase (APX), (**C**) Guaiacol peroxidase GPX, (**D**) Polyphenol oxidase (PPO), and (**E**) phenylalanine ammonia lyase (PAL). The values represented on bars are means ± standard deviation of three replicates (*n* = 3). Different letters indicate statistically significant differences between the treatments as evaluated by ANOVA and Duncan’s multiple comparison test (*P* < 0.05)

Antioxidant enzymes activity plays a crucial role in the growth, development, and detoxification of reactive oxygen species (ROS). In *S. fruticosa* cultures, superoxide dismutase (SOD) activity increased significantly at 1000 mM NaCl culture (Fig. 6A). Different treatments at 700 mM with 0.5% BE, 100 ppm SeNPs, or 50 µM MeJA yielded varying SOD responses, while the 1000 mM NaCl with the same treatments reduced SOD activity compared to saline cultures only (F = 228.57, Fig. 6A).

Other antioxidant enzymes, including ascorbate peroxidase (APX) and guaiacol peroxidase (GPX), exhibited the highest activities in control cultures, which declined significantly (*P* < 0.05) at 700 mM or 1000 mM NaCl. APX activity increased by 49% and 20%, respectively, in 700 mM NaCl-stressed culture supplemented with 0.5% BE or 100 ppm SeNPs, while 50 µM MeJA decreased it by 30%. In 1000 mM media, APX declined with 0.5% BE or MeJA but increased with SeNPs respective to relevant stressed culture (F = 199.24, Fig. 6B). Both 700 mM and 1000 mM cultures showed significantly higher GPX activity with different elicitors compared to saline cultures (F = 615.49).

Polyphenol oxidase (PPO) activity, critical for antioxidant defense, decreased significantly (*P* < 0.05) in the 700 mM and 1000 mM NaCl- stressed cultures relative to the control. However, PPO activity increased in 1000 mM NaCl-treated culture elicited with 0.5% BE, while the highest activity occurred in 700 mM culture supplemented with 100 ppm SeNPs (F = 216.08, Fig. 6D). Conversely, PPO activity declined in 1000 mM cultures supplemented with SeNPs or MeJA compared to their corresponding stressed cultures.

Phenylalanine-ammonia-lyase (PAL), the key enzyme in phenol metabolism, PAL significantly increased in 700 mM-treated cultures and remained unchanged at 1000 mM NaCl. The maximum activity of PAL occurred at 700 mM NaCl culture with 0.5% BE and also increased significantly (*P* < 0.05) with 100 ppm SeNPs, but it was unchanged with 50 µM MeJA compared to stress medium. In 1000 mM cultures, supplementation with 0.5% BE, 100 ppm SeNPs, or 50 µM MeJA significantly stimulated PAL activity compared to stressed cultures (F = 148.31, Fig. 6E).

### Changes in total phenolic content, total flavonoid content, flavonol and DPPH radical scavenging activity of *S. fruticosa* shoot cultures in response to elicitors and/or NaCl

**Fig. 7:**
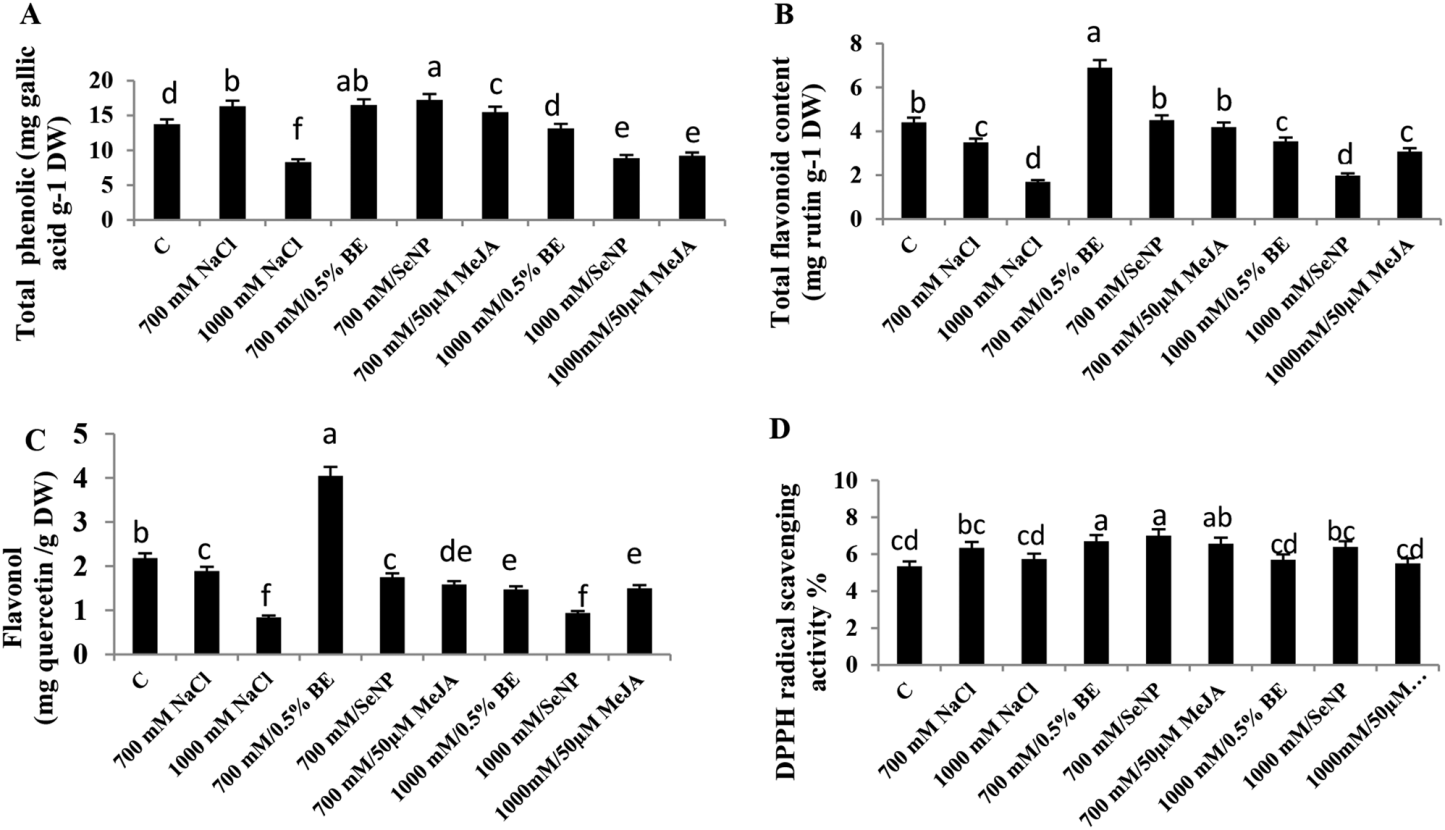
Influence of NaCl treatments (700mM and 1000mM) and their elicitation with 0.5% bacterial extract (BE), 100 ppm selenium nanoparticles (SeNPs) or 50 µM methyl jasmonate (MeJA) on phenolic contents and scavenging activity in *Sarcocornia fruticosa* shoot cultures. Total phenolic content (**A**), Total flavonoid (**B**), Flavonol (**C**), and DPPH free radical scavenging activity (**D**). The values represented on bars are means ± standard deviation of three replicates (*n* = 3). Different letters indicate statistically significant differences between the treatments as evaluated by ANOVA and Duncan’s multiple comparison test (*P* < 0.05)

Total phenolic content in *S. fruticosa* shoot cultures increased significantly (*P* < 0.05) at 700 mM, but declined at 1000 mM NaCl medium ) F = 138.63, Fig. 6A). The addition either BE or SeNPs to saline cultures (700 mM or 1000 mM NaCl) enhanced the phenolic content. Conversely, 50 µM MeJA reduced phenolic content at 700 mM, which increased at 1000 mM NaCl corresponding saline culture (Fig. 7A).

In terms of total flavonoid and flavonol content, a significant decrease was observed (*P* < 0.05) in both NaCl-stressed cultures (Fig. 7B and C). The highest contents of both compounds were observed in the 700 mM NaCl- treated culture with BE, with notable increases at 1000 mM NaCl cultures supplemented with the same elicitor. However, adding 100 ppm SeNPs did not significantly affect flavonoid and flavonol contents, although the flavonoid content increased when 100 ppm SeNPs was added to 700 mM NaCl- stressed culture. Subsequently, 50 µM MeJA induced total flavonoids in both the 700 mM and 1000 mM NaCl- treated cultures. Whereas, MeJA led to a notable decrease in flavonol content at 700 mM NaCl concentration, which increased at 1000 mM NaCl respective to the relevant stress cultures (F = 45.73, Fig. 7C).

DPPH radical scavenging activity increased at 700 mM NaCl medium but changed insignificantly at 1000 mM NaCl culture. Notably, DPPH scavenging activity improved significantly (*P* < 0.05) at 700 mM NaCl medium supplemented with 0.5% BE, 100 ppm SeNPs, or 50 µM MeJA. Conversely, addition of 0.5% BE or 50 µM MeJA at 1000 mM NaCl did not significantly alter DPPH activity, while 100 ppm SeNPs increased the scavenging activity relative to stress cultures (F = 11.34, Fig. 7D).

### Changes in isorhamnetin (IR) contents of *S. fruticosa* shoot cultures in response to elicitors and/or NaCl

**Fig. 8:**
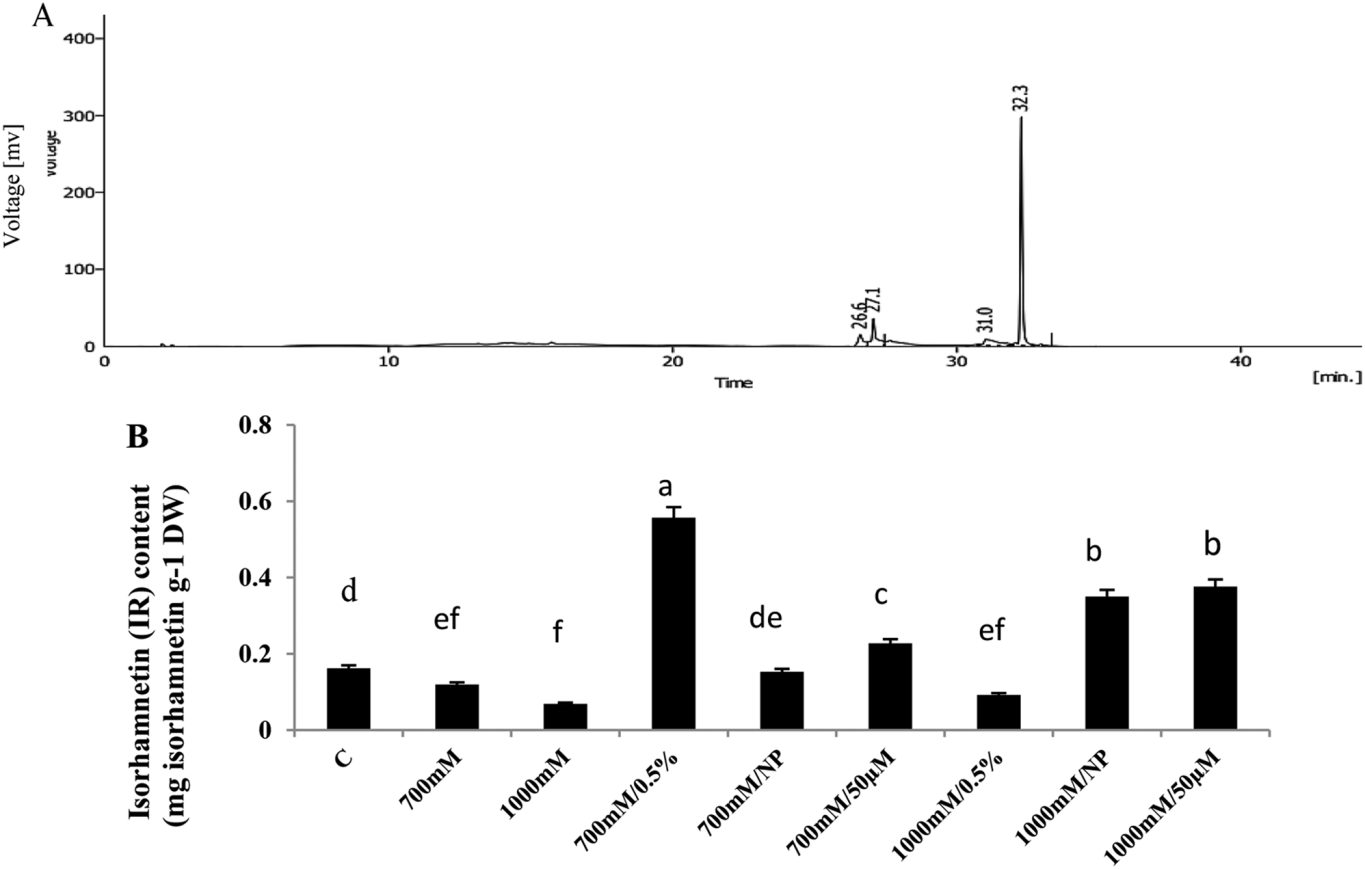
HPLC chromatograms of isorhamnetin (IR) (**A**). Effect of NaCl treatments (700mM and 1000mM) and their interaction with 0.5% bacterial extract (BE), 100 ppm selenium nanoparticles (SeNPs), or 50 µM methyl jasmonate (MeJA) on isorhamnetin (IR) content in *Sarcocornia fruticosa* shoot cultures (**B**). The values represented on bars are means ± standard deviation of three replicates (*n* = 3). Different letters indicate statistically significant differences between the treatments as evaluated by ANOVA and Duncan’s multiple comparison test (*P* < 0.05)

Isorhamnetin was identified by high-performance liquid chromatography (HPLC) using authentic standards (Fig. 8A). In comparison to control culture, isorhamnetin (IR) decreased at the 700 mM or 1000 mM NaCl- stressed culture. In contrast, a significant increase in isorhamnetin content was recorded after supplementation both saline cultures with all elicitors, while the maximum accumulation (4.6-fold) observed with 0.5% BE at 700 mM NaCl culture in respective to the relevant stress culture (F = 72.213, Fig. 8B).

### Hierarchical clustering analysis, principle component analysis, and Pearson correlation coefficient analysis

**Fig. 9:**
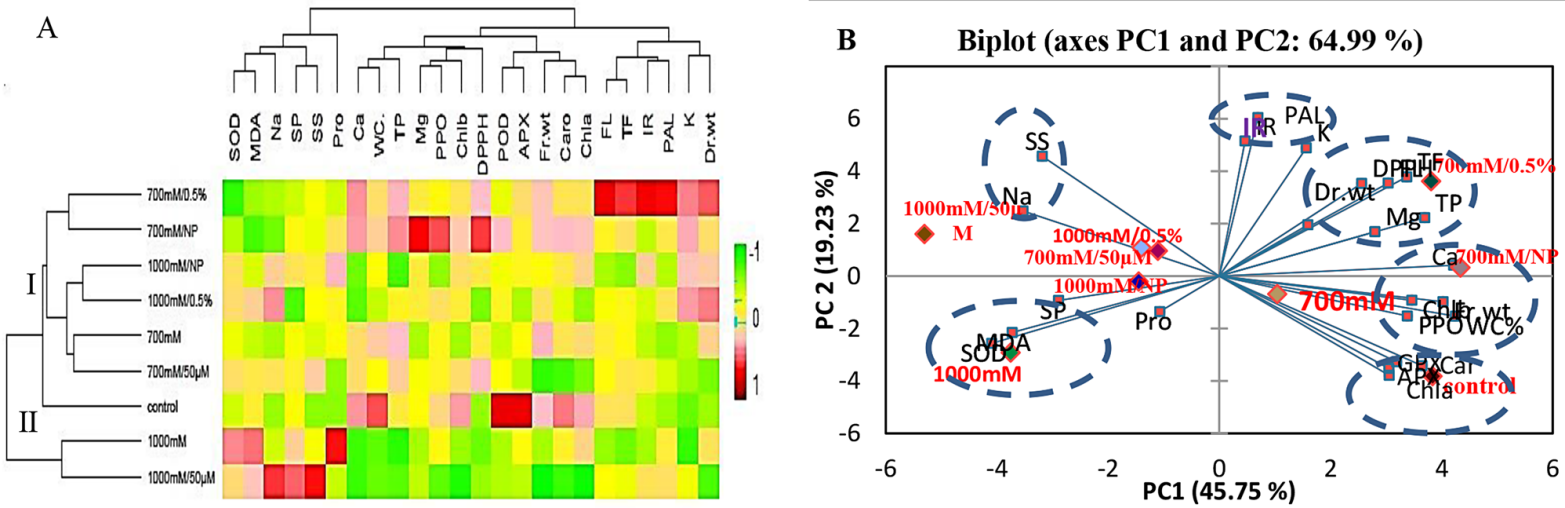
Generated heat map, hierarchical clustering (**A**), and principal component analysis biplot (PCA). (**B**) for various treatments and parameters studies in *S. fruticosa* shoot cultures under NaCl treatments (700mM and 1000mM) and their elicitation with 0.5% bacterial extract (BE), 100 ppm selenium nanoparticles (SeNPs), or 50 µM methyl jasmonate (MeJA). Hierarchical clusters were generated by average linkage method with correlation distance. The mean values of different parameters were normalized and clustered. Color scale showed the intensity of the normalized mean values of different parameters. Biplots’ core lines indicate negative or positive correlations between variables, with closer lines indicating stronger correlation with a specific treatment. The variables included Fr.Wt (shoot fresh weight), Dr.wt (shoot dry weight), WC (water content), Chl a (chlorophyll a), Chl b(chlorophyll b), Caro (carotenoid), Na^+^ (sodium), K^+^ (potassium), Ca^2+^ (calcium ), Mg^2+^ (magnesium), Pro (proline), SS (soluble sugars), SP (soluble protein), MDA (malondialdehyde), SOD (superoxide dismutase), APX (ascorbate peroxidase), GPX (Guaiacol peroxidase), PPO (polyphenol oxidase), PAL (phenylalanine ammonia lyase), TP (total phenolic), TF (total), FL flavonoid (flavonol), DPPH (2,2-diphenyl-1-picrylhydrazyl free radical scavenging activity), and IR (isorhamnetin)

The physiological and biochemical results of the *S. fruticosa* shoot cultures in NaCl media, alongside their interactions with elicitors, were analyzed using heat maps, hierarchical clustering analyses, and principle component analyses (PCAs). Hierarchical clustering revealed two distinct clusters (Fig. 9A): Group I included the control, 700 mM, 700 mM/50 µM MeJA, 700 mM/100 ppm SeNPs, and 700 mM/0.5% BE, 1000 mM/100 ppm SeNPs, 1000 mM/0.5% BE, while Group II included 1000 mM and 1000 mM/50 µM MeJA. It is noted that the applied heat map reflected the trend of the parameters (decreasing or increasing) relative to the matrix code.

Principle component analysis (PCA) was performed to evaluate the relationships between traits under different treatments in shoot cultures of *S. fruticosa.* The two components of PCA (PC1 = 45.75 and PC2 = 19.23) collectively explained 64.98% of the data variability (Fig. 9B), with a clear distinction between treatments such as 700 mM NaCl with elicitors and unelicited 1000 mM NaCl. Moreover, a great distance was observed between chlorophyll, FW, DW and Na^+^ soluble sugars; however, the content of MDA, proline, and SOD were more related to each other. The positive side of PC1 correlated with chlorophyll content and growth-related traits, whereas the negative side correlated with proline and stress indicators. Five associative groups were identified: first group contained the 700 mM NaCl with 0.5% BE which related to phenolic compounds and isorhamnetin content. The second includes the control, unelicited 700 mM NaCl, and 700 mM NaCl with 100 ppm SeNPs, which related to the antioxidant enzymes, FW, and WC%. The third group includes 700 mM NaCl with 50 µM MeJA, 1000 mM NaCl with 0.5% BE, and 1000 mM NaCl with 100 ppm SeNPs. The fourth group includes 1000 mM NaCl which associated with MDA, SOD, soluble proteins, and proline accumulation. The last group contained 1000 mM NaCl with 50 µM MeJA, which related to Na ions and soluble sugars (Fig. 9B).

**Fig. 10:**
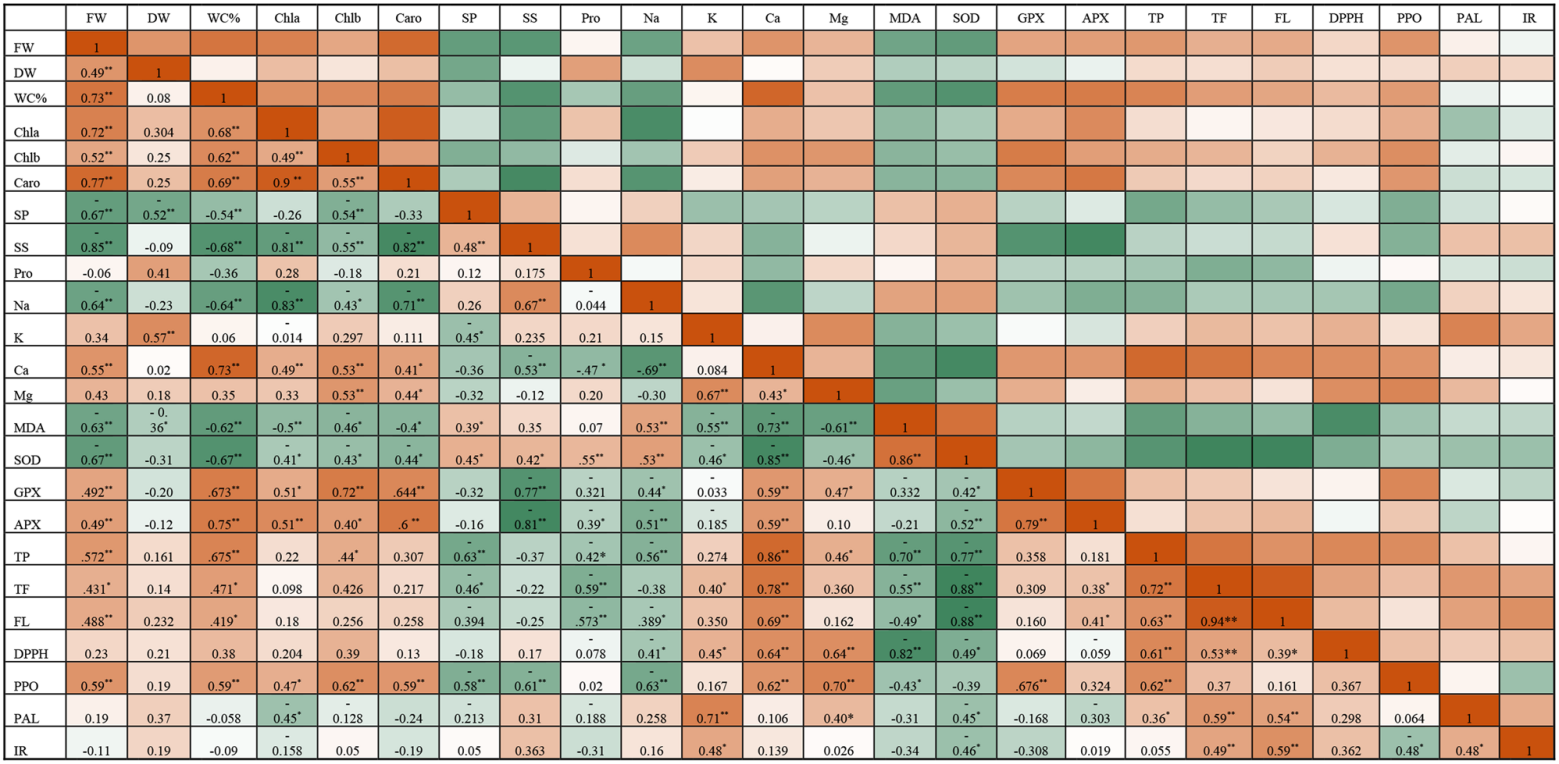
Pearson’s correlation coefficients between pairs of evaluated physiological parameters. *, represented *P* ≤ 0.05; **, represented *P* ≤ 0.01

In addition, the Pearson correlation revealed a strong negative correlation (*p* ≤ 0.05) between the FW, DW, photosynthetic pigments (Chl a, Chl b, and carotenoids) and lipid peroxidation, and Na ion content (Fig. 10). Furthermore, a strong positive relationship was observed between the Mg^2+^ and Ca^2+^ contents and the biomass (FW and DW), photosynthetic pigments, antioxidant enzymes (APX, GPX, and PPO), and phenolic content. Also, a positive correlation was observed between PAL activity and content of total phenolic, total flavonoids, flavonols, and isorhamnetin, especially in shoot cultures at 700 mM NaCl medium supplemented with 0.5% BE. Moreover, positive correlation was observed between soluble sugars, soluble proteins, proline, SOD, and oxidative stress indicators (MDA) in 1000 mM NaCl medium elicited with 50 µM MeJA (Fig. 10).

Color scale shows the intensity of the normalized mean values of different parameters. The variables included Fr.wt (fresh weight), Dr.wt (dry weight), water content (WC%), chlorophyll (Chla, Chlb), carotenoids (Caro), Ca^2+^ (calcium), Mg^2+^ (magnesium ), K^+^ (potassium), Na^+^ (sodium), SP (soluble protein), SS (soluble sugars), Prol (proline), MDA (malondialdehyde), SOD (superoxide dismutase), APX (ascorbate peroxidase), GPX (peroxidase), TP (phenolics ), TF (total flavonoid), FL (favonols), PPO (polyphenol oxidase), PAL (phenylalanine), DPPH (2,2-diphenyl-1-picrylhydrazyl), IR (Isorhamnetin).

## Discussion

Application of plant Tissue Culture (PTC) technology to study halophytic plants can improve their multiplication systems, functional components production, and salt stress resistance. In this study, *S. fruticosa* growth was optimal in the control culture (2% NaCl ~ 320 mM) and efficient at 700 mM NaCl, but disrupted at 1000 mM NaCl. Similar results were reported by [60], who suggest a better performance of perennial Egyptian *S. fruticosa* at 200 mM and 400 mM NaCl. In addition, as discussed by [61], 700 mM NaCl did not significantly inhibit shoot growth. However, increased dry weight (DW) by elevation NaCl levels may suggest an adaptation mechanism allowing *S. fruticosa* culture to grow effectively. Reduction in water content (WC%) under salt treatments (700 mM and 1000 mM) may be attributed to the osmotic challenge that reduces the water uptake and availability.

The ability of the *E. cloacae* extract (0.5% v/v BE) to create numerous cell components may explain the increase in fresh (FW) and dry weight (DW) of *S. fruticosa* shoot cultures under NaCl treatments. Halophytic bacteria can produce various phytoalexin, low-molecular-weight substances, osmoprotectants, lipopolysaccharides, peptidoglycans, and various other cell wall materials released into the culture medium [31]. These components might activate defensive signal transduction pathways and improve physiological performance [62].

Growth stimulation under saline cultures (700 mM and 1000 mM) treated with 100 ppm SeNPs might be attributed to reduce osmotic stress. This enhancement could be due to decline MDA content, increasing proline content, soluble sugars, and antioxidant capacity. Additionally, increased the protein content suggests the role of selenium in promoting the synthesis of cysteine, methionine, and the selenium‒amino acids (Se-Cys and Se-Met), which are incorporated into protein biosynthesis [63].

Biomass reduction of *S. fruticosa* shoots by 50 µM methyl jasmonate (MeJA) at 700 mM or 1000 mM NaCl may be due to unfavorable effects caused by prolonged exposure to MeJA that lead to phytotoxicity and inhibit growth. Methyl jasmonate treatment can reduce plant growth due to delay the production and bio-activities of endogenous gibberellins. Plant gibberellin hormones can interact with MeJA synergistically, inhibiting growth [64]. The optimization of MeJA’s effect was dependent on its concentration, culture exposure time, and the plant growth stage [65].

The reduction in photosynthetic pigment contents in saline cultures (700 mM and 1000 mM) may be consequence of the reduction in Mg^2+^ content, degradation of membrane integrity, increased chlorophyllase activity, which leads to chlorophyll degradation [66]. Positive correlations between FW with WC% (*r* = 0.73^**^) and Chla (*r* = 0.72^**^) confirmed that declining *S. fruticosa* growth can be due to a reduced water uptake, which reduces cell turgor in the plant tissues, consequently reducing the availability of photosynthetic assimilates for growth. Under saline environment (700 mM and 1000 mM) treated cultures, *S. fruticosa* shoots may exhibit reduced stomatal conductance, net photosynthetic rate depletion, and possibly decreased biochemical constraints on photosynthesis, as reported by [67]. However, the significant increase in Chl a/b ratio in the saline medium of *S. fruticosa* culture might be a potential adaptive response to extreme environmental conditions. Growth maintenance under high salinity was accompanied by increased in Chl a/b ratio and phenolic content, consistent with previous studies [68].

The accumulation of Chl b by *E. cloacae* extract (0.5% BE) under NaCl treatments was thought to be due to the importance of Chl b, which has a protective effect on photosystems and decreases the photo-oxidation rate [69]. In contrast, the reduction or insignificant change in Chl a was probably due to a reduction in the open PSII reaction centers (qP) and/or a reduction in the efficiency of excitation capture by the open PSII reaction centers [70]. Photosynthetic pigments increased in *S. fruticosa* salt cultures treated with SeNPs. This increment may be linked to the ability of SeNPs in diminishing the salt stress by alleviating adverse impacts on PSII functioning, reducing chloroplasts damage, and improving photosynthetic capacity by preserving the water-splitting complex and amending ion homeostasis [71]. The greatest reduction in photosynthetic pigments by MeJA elicitation of salt-treated *S. fruticosa* shoot cultures suggested decreased photosynthetic activity and increased chlorosis, that confirmed by the change in nodal color to pale yellow or yellow-brown. Carotenoids play a vital role as non-enzymatic antioxidants in protecting the photosynthetic system; however, a decrease in carotenoids in *S. fruticosa* cultures may have indicated that carotenoids were not available to protect shoot cultures against oxidative damage and did not play a role in salt tolerance [72].

In the present case, increasing K^+^ and K^+^/Na^+^ ratio at 700 mM NaCl culture suggested that *S. fruticosa* can sequester Na^+^ in their vacuoles and maintain a stable level of cytosolic K^+^ as an avoidance mechanism for osmotic homeostasis [73]. Sodium ions increased in the shoots of *S. fruticosa* culture in response to 1000mM NaCl which may be resulted from excess Na^+^ accumulation and deficiency of K^+^. Reduction in the K^+^/Na^+^ ratio was probably related to a mineral imbalance, reduction in the Ca^2+^ and Mg^2+^ contents, and competition among the Na^+^, K^+^, and Ca^2+^ uptake [68].

Along with these findings, increasing NaCl levels decreased the Mg^2+^ content in *S. fruticosa* cultures; Mg^2+^ is essential for protein synthesis and a strong chlorophyll structure [74]. The positive correlations between FW and DW with K^+^ (*r* = 0.57^**^) showed that improving *S. fruticosa* cultures growth attributes can be partly related to an increase in the uptake of essential elements such as potassium ions. While, the negative correlation between Na^+^ content and FW (*r* = − 0.64^**^) and Chla (*r* = − 0.83^**^) can be suggested that the Na^+^ toxicity likely results in a reduction in photosynthesis and, consequently, a decrease in fresh matter.

In this study, the shoots cultured with 0.5% BE under NaCl displayed insignificant difference in Na^+^ content, concomitant with an increase in K^+^ uptake which led to an enhancement of the K^+^/Na^+^ ratio. This result likely relates to the efficiency of the cultures in absorbing K^+^ to maintain osmotic homeostasis. These findings were corroborated by increases in shoot growth and the contents of K^+^, Mg ^2+^, and Ca^2+^ in the *S. fruticosa* cultures, as shown in the PCA analysis.

The positive impact of SeNPs on *S. fruticosa* shoot cultures at 700 mM NaCl may restricted from reduced Na^+^ accumulation and increased K^+^ and Ca^2+^ uptake, which maintained a higher K^+^/Na^+^ ratio. The significant increase in potassium content at 1000mM NaCl medium supplemented with 100 ppm SeNPs could be confirm the culture’s ability to maintain homeostasis for both K^+^ and Na^+^. Consequently, SeNPs improved *S. fruticosa* resistance under saline treatment by enhancing shoot growth, photosynthetic capacity, Mg^2+^ and Ca^2+^ levels, osmotic adjustment, potential antioxidant enzymes. SeNPs upregulated the expression of Na^+^/H^+^ antiport and tonoplast H^+^-ATPase, thereby reducing Na^+^ toxicity and improving salt tolerance [75].

The decrease in the K^+^/Na^+^ ratio of *S. fruticosa* shoot culture treated with MeJA under NaCl may be related to the antagonism between K^+^ and Na^+^ ions content. Additionally, insignificant changes in Ca^2+^ levels may correlate with lower salt tolerance. Nevertheless, our study demonstrated that the restricted Ca^2+^ and K^+^ contents may have contributed to decrease culture growth, as indicated by the Pearson correlation coefficient, which showed a positive relationship between the level of Ca^2+^ accumulation, biomass, and photosynthetic pigments contents, antioxidant enzymes (APX, GPX, and PPO) and phenolic components. This highlights the important role of Ca^2+^ as a second messenger in Na^+^ efflux through the SOS1 signaling pathway via the plasma membrane, promoting salt tolerance [76].

The significant increase in soluble sugars in *S. fruticosa* shoot cultures with rising NaCl levels may have facilitated cellular adaptation to maintain vital activity, mitigate the detrimental effects of salinity, and scavenge hydroxyl radicals to protect plants from oxidative damage [77]. In similar, the elicitation of *S. fruticosa* explants with 0.5% v/v BE, 100 ppm SeNPs, or 50 µM MeJA in the saline media (700 or 1000 mM) increased the amounts of soluble sugars, potentially aiding *S. fruticosa* shoot cultures in overcoming the adverse effect of NaCl while maintaining osmotic adjustment.

Increase in soluble proteins of *S. fruticosa* cultures under saline condition may have affected the cytoplasmic viscosity of the cells, played a significant role in osmotic adjustment, and provided a nitrogen reserve mobilized [78]. Elicitation of shoot explants of *S. fruticosa* with 0.5% BE at saline media (700 or 1000 mM) resulted in decreased the soluble proteins; these results suggest that the proteins were degraded due to increasing protease activity [79]. In a similar vein, the reduction or insignificant change in soluble proteins following the application of SeNPs in both saline media may confirm the significant role of other mechanisms in mitigating salt stress, such as increases in soluble sugars, prolines, and antioxidant enzymes. MeJA elicitation led to the accumulation of soluble proteins in *S. fruticosa* shoot cultures grown in either NaCl media, which may be attributed to the synthesis of stress proteins or proteinase inhibitors that help adjust to osmotic stress [80]. This is supported by the heat map and PCA analysis results, indicating a strong positive relationship among the 1000 mM NaCl cultures elicited with MeJA and the amounts of soluble sugars and soluble proteins.

In addition, maintain osmotic adjustments in *S. fruticosa* shoot cultures may realize through the increase of proline in both saline cultures (700 mM or 1000 mM) and in 700 mM NaCl-treated culture elicited with SeNPs. A negative correlation between proline content and WC% (*r* = − 0.41^*^) suggested that as WC decreased, proline content increased as an osmolyte. Proline retains water (maintaining cell turgor), eliminates excess ions through a dilution effect, stabilizes membrane integrity by preventing electrolyte leakage, and facilitates the transports ROS [81]. The reduction of proline in *S. fruticosa* cultures at 1000 mM NaCl with different elicitors may be compensated by other osmolytes, such as soluble sugars. Furthermore, this reduction may have resulted from rapid degradation, potentially providing sufficient energy to enhance mitochondrial oxidative phosphorylation and ATP generation [82].

The fact that the saline stress in *S. fruticosa* cultures at 700 mM NaCl reduced the level of malondialdehyde (MDA), revealed that no oxidative radicals was generated by NaCl and the antioxidant capacity, along with proline accumulation was increased. Similar results were observed in *S. fruticosa* shoots under salt stress [61]. Conversely, cultures at 1000mM NaCl exhibited higher levels of MDA, attributable to the oxidative stress induced by NaCl. A significant correlation between Na^+^ content and MDA levels (*r* = 0.53^**^) supports the fact that Na^+^ toxicity alters membrane lipids and leading to lipid peroxidation. Shoots of *S*. *fruticosa* activated proline synthesis and increased SOD activity, potentially protecting the culture against ROS generation and stabilized the lipid membranes. A reduction in MDA content with 0.5% BE or SeNPs supplementation suggested their crucial role in alleviate NaCl’s adverse effects, quenching ROS and enhancing the activity of antioxidant enzymes such as GPX and APX. *Enterobacter* extract contains polysaccharides and protein enzymes that may improve growth and stress tolerance. The PCA analysis demonstrated a positive relationship between BE or SeNP treatments and accumulated K^+^ and Ca^2+^, improved the FW and DW, and activated the antioxidant enzymes. Subsequently, the elevation of MDA level with MeJA in 700 mM NaCl-treated culture may be attributed to the production of superoxide (O2^•−^) and hydroxyl radicals (OH) [65].

The significant increase in superoxide dismutase (SOD) activity in *S. fruticosa* cultures with higher levels of NaCl may recommend the role of SOD in alleviation the reactive oxygen species (ROS) damage by dismutation or regulating the appropriate level of superoxide (O_2_^•−^). The observed reduction in activities of ascorbate peroxidase (APX), guaiacol peroxidase (GPX), and polyphenol oxidase (PPO) in saline cultures could be attributed to their roles in regulating H_2_O_2_ level. Moreover, they might have been at a threshold level in the chloroplast and cytosol [83], particularly highlight by the highest MDA level in 1000 mM NaCl-treated culture, and diminished growth parameters.

After elicitation of *S. fruticosa* cultures with 0.5% BE or 100 ppm SeNPs at saline media (700 mM or 1000 mM), SOD activity reduced, which indicates the consumption of this enzyme in alleviating salinity stress through detoxification of the free radical. This reduction may correlate with declined MDA levels and enhance the growth. In contrast, enhanced the activities of APX and GPX under NaCl with 0.5% BE or SeNPs underscore their importance in bolstering enzymatic antioxidant defenses to prevent oxidative damage and enhanced H_2_O_2_ detoxification. However, the reduction in APX at 1000 mM NaCl with either BE or SeNPs, suggesting diminished scavenging of O^2•−^ and implicating this enzymes in the mechanisms of salt tolerance. Notably, increasing SOD and GPX activities in cultures elicited with 50 µM MeJA under salt conditions may contribute as defense mechanism in signal transduction pathways and ROS detoxification. Variations in polyphenol oxidase (PPO) activity in *S. fruticosa* cultures elicited with different elicitors at 700 mM or 1000 mM NaCl highlight its role in antioxidant response against stress through scavenge ROS or maintain the total phenol content. To sum up, these finding revealed the coordination between enzymes activities to maintain the protection against the MDA, H_2_O_2_, and O_2_^•−^ scavenging process in saline media.

The accumulation of phenolic in *S. fruticosa* cultures at 700 mM NaCl could be due to enhanced phenylalanine ammonia-lyase (PAL) activity, along with a decrease in PPO activity, which improved DPPH scavenging activity. Although the phenolic content increased, the total flavonoid content decreased, as observed in [84]. Conversely, reduction in the phenolic and flavonoid contents of *S. fruticosa* cultures in 1000 mM NaCl-treated culture, potentially due to the minimal change of PAL activity and their use as reducing mediators for the PPO enzyme.

Enhancement total phenolic (TP), total flavonoid (TF), flavonol content (FL), as well as DPPH activity at saline media (700 mM or 1000 mM) treated with BE or SeNPs, may result from the activation of the PAL enzyme and synthesis of new phenolic compounds. These findings could contribute to mediate adaptation and amelioration the osmotic impacts of a saline medium. As reported by [85], the vital role of SeNPs is to increase secondary metabolite production, PAL activity, and upregulation of PAL gene expression. Hierarchical clustering analysis, heat map, and PCA revealed a strong positive relationship between 700mM NaCl-treated with 0.5% BE and the TP, TF, FL, and PAL activity. Furthermore, increase the total phenolic content at 1000 mM NaCl- treated cultures with MeJA, presumably from PAL stimulation. However, increasing total flavonoid and flavonol contents in *S. fruticosa* cultures by elicitation both saline cultures with MeJA may relate to its various defense mechanisms. The negative relationship between MeJA elicitation, growth, and antioxidant activity suggests that MeJA relies on non-enzymatic antioxidants to enhance salt tolerance and mitigate harmful effects.

Consequently, the analysis by HPLC indicated a decrease in isorhamnetin content in *S. fruticosa* shoot cultures at NaCl levels (700 or 1000mM NaCl). This reduction may be due to decline in TF and FL content, as well as a shift in energy resources toward protective mechanisms like osmolyte production (soluble sugars and proline). Our finding is supported by Pearson correlation, since a significant positive correlation between isorhamnetin content and total flavonoid and flavonol levels (r = 0.49**, 0.59** respectively). The significant accumulation of isorhamnetin at 700 mM NaCl culture treated with 0.5% BE, potentially due to increased PAL activity and other enzymes involved in flavonoid biosynthesis. It should also be pointed out that the metabolic contents of *Enterobacter* cellular extracts trigger plant defense responses and secondary metabolite production [86]. The elicitation of halophytic plants using bacterial extracts represents a novel approach that has not been previously explored in the literature, potentially offering new insights into enhancing salinity tolerance and secondary metabolites production. Moreover, PCA confirmed the positive relationship between isorhamnetin accumulation and levels of total phenolic and total flavonoid contents. Thus, our findings suggested that isorhamnetin production is closely related to the bacterial strain, elicitation method and the optimization of the culture’s conditions (e.g., nutrition, pH, and temperature). Although 100ppm SeNPs in *S. fruticosa*’s saline culture enhanced biomass production, isorhamnetin accumulation showed variability, possibly reflecting PAL enzyme activity. The impact of SeNPs on distinct species varies based on growth phases, speciation, cultivation circumstances, exposure period, and physiological, biochemical, and molecular processes; however, secondary metabolite modulations in response to nanoparticles are complex and often unpredictable [87]. Finally, the application of 50 µM MeJA at both NaCl levels adversely affected culture growth but positively influenced isorhamnetin production in *S. fruticosa* cultures. Thus, applying MeJA may stimulate free radicals generation and subsequent defense mechanisms in cultured cells, inducing signal transduction followed by the accumulation of isorhamnetin in *S. fruticosa* shoot cultures. Consequently, adjusting different factors such as plant species, culture type and composition, elicitor type and concentrations, and treatment period are crucial for the efficiency of the elicitation strategies and secondary metabolite production [88].

Overall, these results suggest that the saline responses based on ion transport, osmolyte and phenolics accumulation were efficient to reduce oxidative damage. Interestingly, elicitation both saline cultures with BE and SeNPs increase isorhamnetin production with maintain culture biomass. That based on decreasing the oxidative stress by stimulation the activity of antioxidant enzymes and phenolic accumulation. However, methyl jasmonate (MeJA) had a negatively impacted on growth by increasing Na^+^ and MDA accumulation, enhanced isorhamnetin accumulation.

## Conclusion

*Sarcocornia fruticosa* cultures exhibited sustained growth at both 700 and 1000 mM NaCl, with optimal performance at 700 mM. This resilience was attributed to enhanced salt-adaptation processes, including the accumulation of osmoprotectants, potassium ions, and phenolic antioxidants, alongside reduced oxidative stress marker malondialdehyde (MDA). The reduction in enzymes activities at both NaCl concentrations, suggest a shift towards non-enzymatic antioxidant mechanisms. Thus, the result attained to increase the growth and secondary metabolites at the same times by BE and SeNP, that not realize with methyl jasmonate (MeJA) that confirmed our objective. While this study provides insights into the physiological roles of elicitors under salt stress, the precise mechanisms remain unclear. So, further research with *S. fruticosa* and other halophytes is warranted to fully elucidate these processes and optimize cultivation strategies for this promising halophytic species. Our findings highlight the potential for applying these elicitors in innovative culture techniques such as hairy root culture or bioreactor technology to other plants for enhancing its production of valuable traits such as nutrients, oils, and metabolites. The implementation of new technologies to enhance promising halophytes will be the opportunity to upgrade their manipulation and production, aiming to increased production of metabolites, as well as resistance to salt stress.

## Data Availability

All of the data and materials supporting our research findings are contained in the Methods section of the manuscript.

## Declarations

This research has not been previously submitted.
